# Draft Genome Sequence of Psychrotolerant *Clostridium* sp. Strain M14, Isolated from Spoiled Uncooked Venison

**DOI:** 10.1128/MRA.00314-20

**Published:** 2020-04-16

**Authors:** Nikola Palevich, Faith P. Palevich, Paul H. Maclean, Ruy Jauregui, Eric Altermann, John Mills, Gale Brightwell

**Affiliations:** a AgResearch Limited, Grasslands Research Centre, Palmerston North, New Zealand; b Riddet Institute, Massey University, Palmerston North, New Zealand; University of Maryland School of Medicine

## Abstract

*Clostridium* sp. strain M14 was isolated from vacuum-packaged refrigerated spoiled venison, and this report describes the generation and annotation of its 3.9-Mb draft genome sequence.

## ANNOUNCEMENT

The psychrotolerant *Clostridium* sp. strain M14 is a Gram-positive, rod-shaped, spore-forming, and slow-growing obligate anaerobe that is able to grow at temperatures below 4°C. Numerous bacterial species belonging to the genus *Clostridium* have been recognized as causative agents of blown-pack spoilage (BPS) in vacuum-packed meat products (1, 2). Previous studies identified a number of isolates with some similarity to that of nonproteolytic C. botulinum type B using classical biochemical differentiation, 16S rRNA gene restriction fragment length polymorphism (RFLP) pattern analyses, 16S rRNA gene sequencing, and PCR amplification of the internal transcribed spacer (ITS) regions (1, 3). However, none of these isolates were deemed toxigenic, and M14 clustered within meat-associated psychrotolerant *Clostridium* amplified 16S ribosomal DNA restriction analysis (ARDRA) group 7 (4).

Strain M14 was originally isolated from a fully blown pack of vacuum-packaged refrigerated spoiled venison nearly 20 years ago (4), freeze-dried, and cultured anaerobically at 10°C in prereduced peptone-yeast extract-glucose-starch (PYGS) broth. Genomic DNA was extracted using a modified phenol-chloroform procedure as previously described (5). A DNA library was prepared using the Illumina TruSeq Nano method and sequenced on the Illumina MiSeq platform with the 2 × 250-bp paired-end (PE) reagent kit v2, producing a total of 3,125,724 PE raw reads. The raw reads were trimmed and assembled using the A5-miseq pipeline v20169825 (6). The *de novo* assembly of M14 produced 36 scaffolds with 184× coverage and an *N*_50_ value of 757,921 bp, with the largest scaffold being 1,669,648 bp long. The draft genome sequence is composed of 3,986,879 bp, with a G+C content of 27.1%. A total of 3,717 putative genes were predicted, along with 81 tRNAs, 19 rRNAs, and 170 noncoding RNAs (ncRNAs), using GAMOLA2 (7). Final gene ontology terms and annotations were assigned to each protein using Diamond v0.9.21.122 (8) and InterProScan v5.36-75.0 (9) to search the NCBI nr database, with the resulting protein set imported into Blast2GO implemented in the OmicsBox software package v1.1.164 (10). All bioinformatics analyses were performed using default settings.

Carbohydrate-active enZYme (11) profiling was analyzed using dbCAN2 (12). The M14 genome was predicted to encode 44 glycoside hydrolases (GHs), 27 glycosyl transferases (GTs), 10 carbohydrate esterases (CEs), and 19 carbohydrate-binding protein module (CBM) families but no polysaccharide lyases (PLs). Overall, approximately 2.7% of the M14 genome (100 CDSs) was predicted to encode either secreted or intracellular proteins dedicated to carbohydrate degradation. The nontoxigenic status of M14 was confirmed, as none of the genes encoding the known deadly neurotoxins of group II C. botulinum (BoNTs) were identified with a search of the whole-genome sequence (WGS) database at NCBI (visited March 2020). A whole-genome alignment to the group II C. botulinum genomes revealed a low sequence homology of <20% similarity to M14. The *Clostridium* sp. strain M14 genome is a valuable resource for future studies investigating the bacterial genetic mechanisms associated with BPS.

### Data availability.

The genome sequence data for *Clostridium* sp. strain M14 were deposited under GenBank accession number JAAMNF000000000, BioProject accession number PRJNA574489, and Sequence Read Archive (SRA) accession number SRR11113219.
